# Peripapillary Vessel Density as Early Biomarker in Multiple Sclerosis

**DOI:** 10.3389/fneur.2020.00542

**Published:** 2020-06-17

**Authors:** Gilda Cennamo, Antonio Carotenuto, Daniela Montorio, Maria Petracca, Marcello Moccia, Antonietta Melenzane, Fausto Tranfa, Anna Lamberti, Antonio L. Spiezia, Giuseppe Servillo, Marcello De Angelis, Martina Petruzzo, Chiara Criscuolo, Roberta Lanzillo, Vincenzo Brescia Morra

**Affiliations:** ^1^Eye Clinic, Public Health Department, University of Naples Federico II, Naples, Italy; ^2^Department of Neurosciences, Reproductive and Odontostomatological Sciences, Federico II University, Naples, Italy

**Keywords:** vascular pathology, biomarker, angio-optical coherence tomography, clinically isolated syndrome, vessel density (VD)

## Abstract

**Background:** To evaluate retinal vessel density (VD) in macular and in peripapillary regions in patients with recent onset of multiple sclerosis, at initial demyelinating event (IDE) and in matched relapsing-remitting multiple sclerosis (RRMS) patients.

**Methods:** We evaluated VD in superficial capillary plexus, deep capillary plexus, choriocapillaris and radial peripapillary capillary plexus in IDE, RRMS patients and in matched healthy controls (HCs) through Optical Coherence Tomography Angiography (OCT-A). Clinical history, including history of optic neuritis, Expanded Disability Status scale and disease duration of patients were collected.

**Results:** Thirty patients (20 with IDE and 10 with RRMS) and 15 HCs were enrolled. IDE patients showed a lower VD in radial peripapillary capillary plexus compared with controls (coeff. β = −3.578; *p* = 0.002). RRMS patients displayed a lower VD in both superficial capillary plexus and radial peripapillary capillary plexus compared with HCs (coeff. β = −4.955; *p* = 0.002, and coeff. β = −7.446; *p* < 0.001, respectively). Furthermore, RRMS patients showed a decreased VD in radial peripapillary capillary plexus compared with IDE patients (coeff. β = −3.868; *p* = 0.003).

**Conclusions:** Peripapillary region vessel density reduction, revealed through OCT-A, might be considered as an early event in MS, and might be relevant as a biomarker of disease pathology.

## Introduction

Multiple sclerosis (MS) is characterized by inflammation, demyelination and axonal loss throughout the central nervous system. Markers for disease pathology are highly needed. The introduction of the optical coherence tomography (OCT), fast and non-invasive imaging technique, allowed to investigate and monitor the structural retinal damages, in particular the ganglion cell and retinal nerve fiber layers in neurological diseases. Previous studies, analyzing the different disease stages often accompanied by optic neuritis, demonstrated that the retinal changes reflect not only the neurodegenerative processes but also the inflammatory disease activity. Since the strong association described between the retinal impairment and brain atrophy on MRI, the OCT parameters play a significant role as biomarkers for MS diagnosis and follow up (1–3). Several reports also described cerebral hypo-perfusion and vascular pathology as pathological changes underpinning MS etiology and evolution (4, 5). Besides the application of advanced MRI techniques such as arterial spin-labeling, optical coherence tomography angiography (OCT-A) offers the unique opportunity to assess integrity of brain vasculature by looking at vascular networks within the retina (6, 7). We recently described a reduction of retinal vessel density (VD) on OCT-A in MS compared with controls. Reduced VD was associated with higher disability, as measured with Expanded Disability Status Scale (EDSS) (8), and was further confirmed over the 1-year follow-up, suggesting VD is a marker of disability, with improved vascularization being inversely associated with lower disability accrual. However, the inclusion of very early cases of MS would have allowed a better understanding about implication of retinal vasculature changes in the disease pathogenesis. Feucht et al. recently studied retinal vasculature network in patients with clinically isolated syndrome (CIS), and found vessel rarefaction of superficial and deep retinal plexus only in eyes suffering from previous optic neuritis, while a higher VD in choriocapillaris layer was associated with recent relapses and MRI activity (9).

The aim of this study is to investigate VD in macular and peripapillary regions in patients with an initial demyelinating event (IDE), namely patients experiencing the first neurologic symptom referable to demyelination in the central nervous system regardless they meet MS or CIS diagnosis at MRI scan according with 2017 McDonald criteria (10). We also aimed to compare vascular changes in the retina between controls, IDE and relapsing-remitting MS (RR-MS) patients through OCT-A.

## Methods

In this cross-sectional study, we enrolled IDE patients and healthy controls (HCs) at the MS Center of the University of Naples “Federico II,” from January 2018 to June 2019. “IDE (initial demyelinating event) was defined as the first neurologic symptom referable to demyelination in the central nervous system, lasting for at least 48 h, regardless patients met RR-MS or CIS diagnosis according with 2017 McDonald criteria (10). We excluded patients with any history of optic neuritis, in order to avoid a bias related to optic nerve direct damage. Family history, motor disability assessed through EDSS, disease duration and previous relapses were recorded for all patients. HC presented with normal neurological and ophthalmic examinations.

Exclusion criteria were (i) a relapse and/or corticosteroid use in the previous month (ii) the presence of systemic vascular diseases (high blood pressure, diabetes, and heart diseases), (iii) clinically relevant lens opacities, (iv) low-quality images obtained with Spectral Domain (SD)-OCT and OCT-A, (v) myopia >6 diopters, (vi) history of intraocular surgery, vitreoretinal, and retinal vascular diseases, uveitis, congenital eye disorders.

Each subject underwent evaluation of best-corrected visual acuity according to the Early Treatment of Diabetic Retinopathy Study (11), slit-lamp biomicroscopy, fundus examination For each subject, we also assessed the mean deviation and pattern standard deviation as measures of visual field for subject with visual fixation above 20%. Finally, we performed both SD-OCT and OCT-A. Ophthalmological evaluation was blinded to subjects' clinical status. The study was approved by the Institutional Review Board of the University of Naples “Federico II” and all investigations adhered to the tenets of the Declaration of Helsinki (protocol number: 142/19). Written informed consents were obtained from the subjects enrolled in the study. The data that support the findings of this study are available from the corresponding author upon reasonable request.

### SD-OCT

The retinal nerve fiber layer and ganglion cell complex thickness were obtained with SD-OCT (software RTVue XR version 2017.1.0.151, Optovue Inc., Fremont, CA, USA). The acquisition protocol for optic nerve head map was used to calculate the retinal nerve fiber layer thickness and it was based on measurements around a circle 3.45 mm in diameter centered on the optic disc. The ganglion cell complex thickness was obtained centering the scan 1-mm temporal to the fovea and covering a 7 × 7 mm area over the macular region. The ganglion cell complex thickness included the measurements from the internal limiting membrane to the outer boundary of the inner plexiform layer (12). Each OCT scan was analyzed in alignment following APOSTEL recommendations and applying the OSCAR-IB protocol for quality control (13, 14). These guidelines were adapted for our device.

### OCT-A

**Figure 1:**
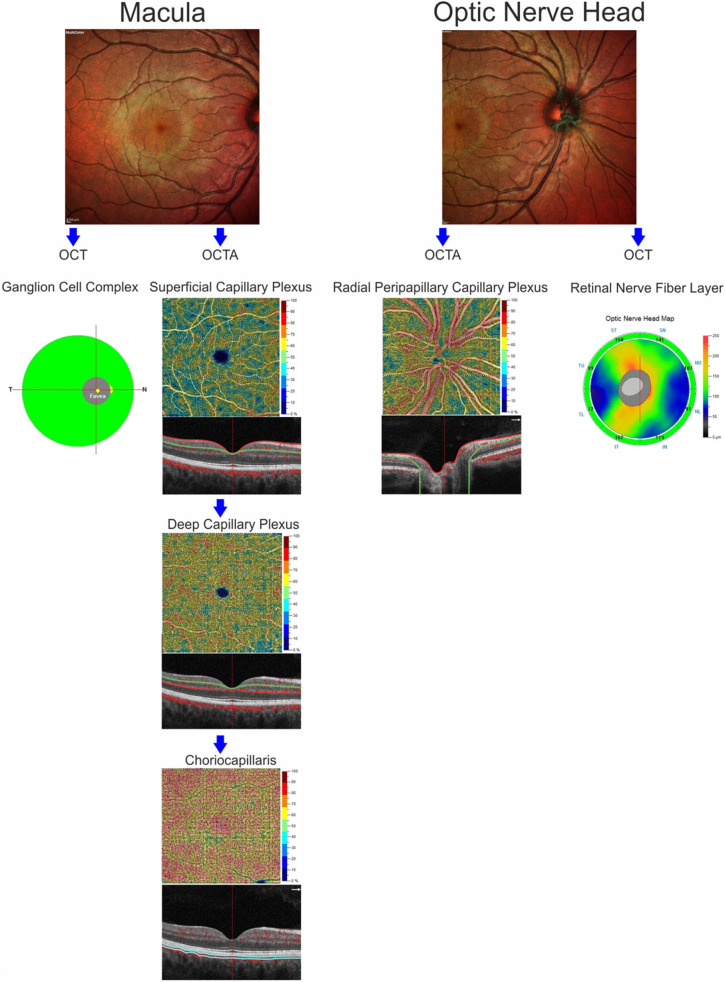
Anatomic illustration of macular and optic nerve head regions showing the retinal structures analyzed by optical coherence tomography (ganglion cell complex and retinal nerve fiber layer) and the retinal and choriocapillaris vascular networks evaluated by OCT-Angiography.

OCT-A images were performed using the RTVue XR Avanti, Optovue, Inc. (software RTVue XR version 2017.1.0.151 Freemont, California, USA) following a standardized protocol based on the split-spectrum amplitude de-correlation algorithm, as previously described (15). Macular capillary plexus was visualized performing a 6 × 6 mm scan over the macular region and the percentage area occupied by the large vessels and microvasculature in the analyzed region defined the vessel density (16). The software identified the VD in whole area of the macular scan considering the two retinal vascular networks, namely the superficial and deep capillary plexuses, and choriocapillaris. The Angio-Vue disc mode automatically segmented the radial peripapillary capillary plexus VD analyzing the whole papillary region with a scanning area of 4.5 × 4.5 mm. VD for the radial peripapillary capillary plexus was analyzed in the superficial retinal layers and extended from the inner layer membrane to the retinal nerve fiber layer posterior boundary (17). From the analysis were excluded the images with a signal strength index <40 and residual motion artifacts. A summary of measures evaluated through SD-OCT and OCT-A is reported in Figure 1.

### Statistical Analysis

Statistical analysis was performed with the Statistical Package for Social Sciences (Version 20.0 for Windows; SPSS Inc., Chicago, Ill, USA). One-way analysis of variance followed by Bonferroni *post hoc* analysis was used to evaluate differences in visual field parameters, age and best-corrected visual acuity among HCs, IDE and RR-MS patients. Chi-squared test was used to determine sex differences among groups. Linear mixed models, including subject, age and sex as covariates, was used to evaluate VD differences in each retinal vascular network (superficial capillary plexus, deep capillary plexus, radial peripapillary capillary plexus) and in choriocapillaris, using group as factor of interests. The same model was used to analyze differences in structural OCT parameters (ganglion cell complex average and retinal nerve fiber layer average) among the groups. Correlations between SD-OCT and OCTA parameters were assessed using linear mixed model for both IDE and RR-MS. Moreover, we analyzed the correlations between best-corrected visual acuity, mean deviation and pattern standard deviation, neurological (EDSS, annualized relapse rate, and disease duration) and OCT-A parameters. Since we evaluated VD in four different regions as dependent variables for the linear mixed models, to correct analysis for multiple regressions, we set the *p*-value for significance at *p* = 0.05/4 (0.012).

## Results

### Demographic and Clinical Features

Thirty patients (20 with IDE and 10 with RR-MS) for a total of 60 eyes and 15 HCs for a total of 30 eyes, were enrolled. There were no significant differences for age, sex, best-corrected visual acuity, and visual field parameters in the three groups. After MRI evaluation, 16 out of 20 IDE (80%) patients met criteria for CIS whereas 4 IDE patients met MRI criteria for RR-MS. Demographic, clinical and OCT features are summarized in Table 1.

**Table 1:** Demographic and clinical characteristics of IDE, RRMS patients and healthy controls.

	**Control**	**IDE**	**RR-MS**
Eyes (N.)	30	40	20
Age, mean ± SD (years)	28.2 ± 8.6	28.9 ± 9.5	29.7 ± 6.3
Sex (female/male)	10/5	10/10	7/3
EDSS, mean ± SD (Range)	-	1.85 ± 0.95 (0–2.5)	2.3 ± 0.57 (1.5–3.5)
Annualized relapse rate, mean ± SD	-	-	0.99 ± 1.05
Disease duration, mean ± SD (years)	-	1.7 ± 2.3	4 ± 1.3
**Onset modality**			
Brainstem, N. (%)	-	6 (30%)	4 (40%)
Pyramidal, N. (%)	-	5 (25%)	2 (20%)
Cerebellar, N. (%)	-	1 (5%)	1 (10%)
Sensory, N. (%)	-	7 (35%)	3 (30%)
Bowel/Bladder, N. (%)	-	0 (0%)	0 (0%)
Cerebral, N. (%)	-	1 (5%)	0 (0%)
**OCT-A parameters (%)**			
Superficial capillary plexus, mean ± SD	53.63 ± 2.53	50.43 ± 4.47	48.75 ± 4.41
Deep capillary plexus, mean ± SD	55.97 ± 4.86	55.15 ± 6.53	53.25 ± 7.06
Choriocapillaris, mean ± SD	74.10 ± 2.66	74.08 ± 2.34	74.15 ± 2.54
Radial peripapillary plexus, mean ± SD	53.23 ± 3.35	49.62 ± 2.90	45.9 ± 3.93
**OCT parameters (μm)**			
Ganglion cell complex average, mean ± SD	100.2 ± 6.79	98.61 ± 9.89	89.54 ± 9.85
Retinal nerve fiber layer average, mean ± SD	103.1 ± 8.19	101.83 ± 10.88	95.15 ± 13.18
**Visual Field parameters**			
Mean Deviation, mean ± SD	−0.51 ± 1.18	−0.59 ± 1.61	−1.19 ± 2.09
Pattern standard deviation, mean ± SD	2.1 ± 0.46	2.42 ± 1.12	2.25 ± 0.83
**Best-corrected visual acuity, mean** **±** **SD (logMAR)**	0.03 ± 0.04	0.02 ± 0.04	0.01 ± 0.03

*IDE, Initial Demyelinating Event; RRMS, Relapsing–Remitting Multiple Sclerosis; EDSS, Expanded Disability Status Scale; OCT-A, Optical Coherence Tomography Angiography; logMAR, logarithm of the minimum angle of resolution; SD, Standard Deviation*.

### SD-OCT

At SD-OCT exam, RR-MS patients showed lower ganglion cell complex values compared with IDE patients (89.54 ± 9.85 vs. 98.61 ± 9.89; *p* = 0.017) and HCs (89.54 ± 9.85 vs. 100.2 ± 6.79; *p* = 0.006). Ganglion cell complex thickness was not different between IDE group and HCs. Retinal nerve fiber layer did not differ between HCs, IDE, and RR-MS patients.

### OCT-A

**Figure 2:**
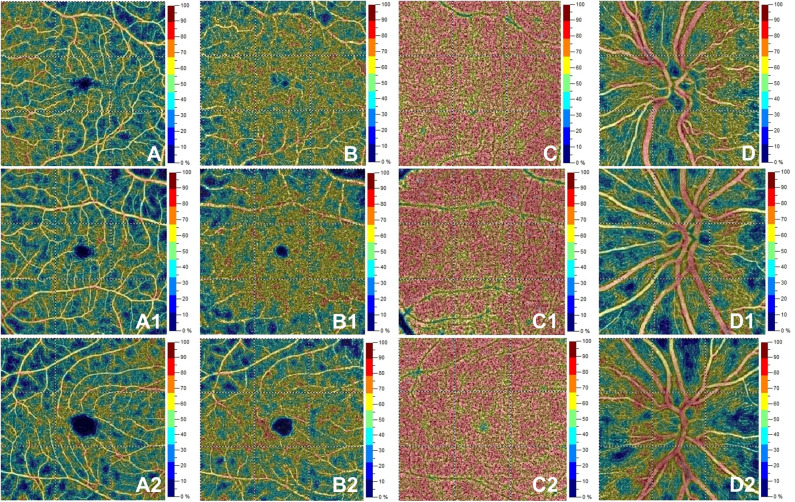
Optical coherence tomography angiography (OCT-A) images from a healthy subject' s left eye (male, 28 years) in the first row show normal vessel density in superficial capillary plexus **(A)**, deep capillary plexus **(B)**, choriocapillaris **(C)** and radial peripapillary capillary plexus **(D)**. The second row depicts OCTA features in the left eye for a patient (female, 28 years) with initial demyelinating event. OCTA reveals normal vessel density in superficial capillary plexus **(A1)**, deep capillary plexus **(B1)**, choriocapillaris **(C1)** with a decrease for vessel density in the radial peripapillary capillary plexus **(D1)**. The bottom row shows a patient's right eye (male, 29 years) affected by relapsing-remitting multiple sclerosis. Here, vessel density is reduced in the superficial capillary plexus **(A2)** and radial peripapillary capillary plexus **(D2)** without vessel density changes in the deep capillary plexus **(B2)** and choriocapillaris **(C2)**.

The VD in radial peripapillary capillary plexus was significantly lower in IDE group compared with HCs (coeff. β = −3.578; *p* = 0.002). VD for both superficial capillary plexus and radial peripapillary capillary plexus was lower for RR-MS patients compared with HCs (coeff. β = −4.955; *p* = 0.002, and coeff. β = −7.446; *p* < 0.001, respectively; see Table 2). RR-MS patients showed a lower VD in radial peripapillary capillary plexus compared with IDE patients (coeff. β = −3.868; *p* = 0.003; see Table 2). The VD in choriocapillaris and deep capillary plexus did not differ between the three groups (see Figure 2). There were no significant correlations between OCT-A measures and visual field parameters (mean deviation and pattern standard deviation) while VD in the deep capillary plexus showed a significant correlation with best-corrected visual acuity (coeff. β = −0.002; *p* = 0.007; Table 3). No correlation was found between OCT-measures and neurological parameters (EDSS, annualized relapse-rate and disease duration).

**Table 2:** Differences in OCTA parameters among IDE, RRMS and control groups.

**OCT-A parameters**	**IDE vs. control**		
	**β**	**(95% CI)**	***P*-value**
Superficial capillary plexus	−3.180	(−5.696 to −0.664)	0.015
Deep capillary plexus	−0.534	(−4.021 to 2.952)	0.758
Choriocapillaris	−0.111	(−1.518 to 1.296)	0.874
Radial peripapillary capillary plexus	−3.578	(−5.724 to −1.431)	0.002
	**RRMS vs. control**		
	**β**	**(95% CI)**	***P*****-value**
Superficial capillary plexus	−4.955	(−7.933 to −1.977)	0.002
Deep capillary plexus	−2.996	(−7.122 to 1.131)	0.15
Choriocapillaris	0.129	(−1.536 to 1.794)	0.877
Radial peripapillary capillary plexus	−7.446	(−9.906 to −4.986)	<0.001
	**RRMS vs. IDE**		
	**β**	**(95% CI)**	***P*****-value**
Superficial capillary plexus	−1.775	(−4.361 to 1.080)	0.216
Deep capillary plexus	−2.461	(−6.418 to 1.496)	0.216
Choriocapillaris	0.240	(−1.357 to 1.837)	0.763
Radial peripapillary capillary plexus	−3.868	(−6.289 to −1.448)	0.003

*IDE, Initial Demyelinating Event; RR-MS, Relapsing–Remitting Multiple Sclerosis; OCT-A, Optical Coherence Tomography Angiography; CI, Confidence Interval*.

**Table 3:** Correlations between vessel density, visual field, and visual acuity for MS patients.

**Regions**		**Mean deviation**	
	**β**	**(95% CI)**	***P*-value**
Superficial capillary plexus	0.079	(−0.023 to 0.182)	0.127
Deep capillary plexus	0.035	(−0.016 to 0.087)	0.173
Choriocapillaris	−0.046	(−0.189 to 0.097)	0.517
Radial peripapillary capillary plexus	−0.103	(−0.200 to −0.005)	0.038
		**Pattern standard deviation**	
	**β**	**(95% CI)**	***P*****-value**
Superficial capillary plexus	−0.018	(−0.098 to 0.062)	0.654
Deep capillary plexus	−0.015	(−0.058 to 0.028)	0.480
Choriocapillaris	−0.001	(−0.116 to 0.113)	0.978
Radial peripapillary capillary plexus	0.017	(−0.057 to 0.092)	0.637
		**Best-corrected visual acuity**	
	**β**	**(95% CI)**	***P*****-value**
Superficial capillary plexus	0.003	(−0.0001 to 0.006)	0.061
Deep capillary plexus	−0.002	(−0.005 to 0.0008)	0.007
Choriocapillaris	−0.0008	(−0.005 to 0.004)	0.733
Radial peripapillary capillary plexus	−0.0002	(−0.003 to 0.002)	0.855

*CI, confidence interval*.

### OCT-A Correlates to SD-OCT

In patients, ganglion cell complex thickness was associated with VD in superficial capillary plexus and radial peripapillary capillary plexus (coeff. β = 1.474; *p* < 0.001 and coeff. β = 1.101; *p* < 0.001). Similarly, retinal nerve fiber layer thickness was associated with VD in radial peripapillary capillary plexus (coeff. β = 0.817; *p* = 0.009; Table 4).

**Table 4:** Correlations between OCTA and OCT parameters in the group including IDE and RRMS patients.

**OCT-A parameters**	**Ganglion cell complex average**			**Retinal nerve fiber layer average**		
	**β**	**(95% CI)**	***P*-value**	**β**	**(95% CI)**	***P*-value**
Superficial capillary plexus	1.474	(0.910 to 2.039)	<0.001	0.486	(0.138 to 1.389)	0.123
Deep capillary plexus	−0.215	(−0.549 to 0.118)	0.197	−0.145	(−0.442 to 0.152)	0.321
Choriocapillaris	−0.166	(−0.983 to 0.649)	0.683	0.712	(−0.142 to 1.567)	0.098
Radial peripapillary plexus	1.101	(0.591 to 1.612)	<0.001	0.817	(0.218 to 1.416)	0.009

*OCT-A, Optical Coherence Tomography Angiography; CI, Confidence Interval*.

## Discussion

Notwithstanding the many progresses achieved over the last years in uncovering different mechanisms contributing to tissue damage and clinical disability in MS, a full understanding of the disease pathogenesis is still hampered by the impossibility to study the premorbid stages of the disease. To overcome this obstacle, a valuable opportunity is provided by the exploration of pathology abnormalities occurring in very early disease phases. Specifically, we hereby investigated the role of vascular abnormalities in MS pathogenesis. We evaluated their role as early marker of disease, analyzing retinal and choriocapillaris VD in patients with recent onset of their first demyelinating episode. OCT-A is a reliable marker of disease and disability accrual in definite MS (8, 15, 18), especially in later stages. However, its role in earlier disease stages is less clear. To our knowledge, only two studies exploring VD variations enrolled early-stage MS patients, but in both cases these were considered in a pooled analysis including also RR-MS patients, making impossible to draw specific conclusions to early stage MS (9, 18). In the present study, the comparison of patients with IDE, RR-MS, and HCs in terms of retinal VD suggests an early involvement of the radial peripapillary capillary plexus, regardless of the presence of retinal atrophy or ongoing inflammation. Feucht and colleagues recently reported a reduced VD of the superficial capillary plexus and deep capillary plexus in eyes of MS and CIS patients affected by optic neuritis, with no changes in the healthy eye (9). In addition, Murphy et al. described a reduction of superficial capillary plexus in eyes affected by optic neuritis and, to a lesser extent, in the healthy eye (18). In our sample, we identified a rarefaction of radial peripapillary capillary plexus both in IDE and RRMS with no history of optic neuritis. Both previous studies (9, 18) described an association between inner retinal layer volumes and density of both the superficial and deep vascular plexuses, suggesting a relationship between retinal atrophy and the consequent reduction in vascularization, induced by the reduced metabolic request of the atrophic layers. In our study, similar associations were identified between OCT-A and structural-OCT parameters in the entire patients' group but, as no retinal atrophy was present in IDE patients, VD reduction in radial peripapillary capillary plexus should not be ascribed to macroscopic structural abnormalities of the retina nor to the presence of optic nerve atrophy. Radial peripapillary capillary plexus rarefaction could be indeed the proxy of a more diffuse vascular involvement in MS pathogenesis or, alternatively, it might be related to subtle microstructural changes of the optic nerve fibers, which might explain the selective VD reduction in radial peripapillary capillary plexus rather than in all the explored vascular districts. Fibers within the retinal nerve fiber layer might suffer indirectly from vascular damage of the optic nerve, in the frame of a more diffuse white matter microstructural damage, that has been described as an early finding in CIS patients (19, 20). As per the insight gained from RR-MS patients, later on in the disease course, radial peripapillary capillary plexus rarefaction increased, with superficial capillary plexus showing VD reduction too, mirroring the development of atrophy in retinal nerve fiber layer and ganglion cell complex. Finally, similarly to what reported by Feucht et al. for CIS/MS patients (9), abnormalities in choriocapillaris VD were not detected in our IDE/RRMS patients. Unfortunately, no formal analysis of the association between choriocapillaris VD and previous relapse rate could be performed, as the majority of our sample was constituted by IDE subjects for whom, by definition, no past disease activity is present in terms of more than one relapse. Furthermore, the lack of correlation between OCT-A and clinical parameters, which might seem counterintuitive, considering previous reports in MS (8, 15, 18), might be similarly accounted for by the mild clinical status of IDE patients. Eventually, due to the cross-sectional nature of the study, we cannot completely rule out that changes in VD without structural-OCT abnormalities might depend on the lower inter-subject variability of OCT-A measures compared with structural OCT measures. When analyzed over the follow-up, SD-OCT shows high level of sensibility for detecting retinal structural changes (21, 22). Longitudinal studies with a larger sample size are highly needed to evaluate the sensitivity for OCT-A in detecting progressive VD loss over the disease course regardless of the inter-subject variability and to assess the contribute of this technique to the already validated standard-OCT. In addition, it is worthy to mention that OCT-A might be more sensitive than SD-OCT in detecting retinal abnormalities associated with subclinical optic neuritis. When compared with visual evoked potentials, SD-OCT was shown to be less sensitive in detecting subclinical optic neuritis (23, 24). To explore this hypothesis a multimodal assessment of optic nerve through SD-OCT, OCT-A, visual evoked potentials and, eventually, MRI scans might shed further lights on this topic. In conclusion, our data suggest that retinal vascular abnormalities are possibly driven by primary vessel involvement, or secondary to structural damage ongoing in the retina and optic nerve during the disease course. The role played by each mechanism seems to differ according to the disease stage, with VD being the proxy of primary vessel involvement or subclinical white matter macrostructural abnormalities in an early stage, and retinal atrophy in a later stage. Regardless of the causative mechanism, our results confirm the relevant role of retinal VD as a non-invasive, early biomarker of disease, independently from the presence of inflammation, although we recognize that the applications of radial peripapillary capillary plexus VD measurements as diagnostic marker in clinical settings will require further studies to explore the specificity of such vessel density rarefaction.

## Data Availability Statement

The datasets generated for this study are available on request to the corresponding author.

## Ethics Statement

This study, involving human participants, was reviewed and approved by the Institutional Review Board of the University of Naples Federico II and all investigations adhered to the tenets of the Declaration of Helsinki (protocol number: 142/19). The patients/participants provided their written informed consent to participate in this study.

## Author Contributions

Conception and design of the study: GC, AC, RL, and VB. Data acquisition: RL, DM, AM, FT, GC, AS, MD, CC, MP, AL, MM, and MP. Data analysis: AC, DM, MP, and MM. Manuscript drafting: AC, GC, DM, MP, GS, VB, RL, and CC. All authors contributed to the article and approved the submitted version.

## Conflict of Interest

RL and VB received personal compensations for speaking or consultancy from Biogen, Teva, Genzyme, Merck, Novartis, Roche and Almirall. MM received research grants from the ECTRIMS-MAGNIMS, United Kingdom and Northern Ireland MS Society and Merck, and honoraria form Biogen, Genzyme, Merck, and Roche. AC research grants from ALMIRALL, and honoraria form Novartis, Merk, Merck, and Biogen. The remaining authors declare that the research was conducted in the absence of any commercial or financial relationships that could be construed as a potential conflict of interest.

The reviewer LL declared a past co-authorship with one of the authors MM, RL to the handling editor.
